# Curcumin Attenuates Fumonisin B1-Induced PK-15 Cell Apoptosis by Upregulating miR-1249 Expression to Inhibit the IRE1/MKK7/JNK/CASPASE3 Signaling Pathway

**DOI:** 10.3390/antiox14020168

**Published:** 2025-01-30

**Authors:** Jia Chen, Dongwei Xiong, Miao Long

**Affiliations:** 1 Key Laboratory of Livestock Infectious Diseases, Ministry of Education, and Key Laboratory of Ruminant Infectious Disease Prevention and Control (East), Ministry of Agriculture and Rural Affairs, College of Animal Science and Veterinary Medicine, Shenyang Agricultural University, 120 Dongling Road, Shenyang 110866, China; 2020200157@stu.syau.edu.cn (J.C.); 2022200183@stu.syau.edu.cn (D.X.); 2 College of Laboratory Animal Medicine, Liaoning University of Traditional Chinese Medicine, 79 Chongshan Road, Shenyang 110847, China

**Keywords:** FB1, Cur, IRE1/MKK7/JNK/CASPASE3, *Ern1*, miR-1249

## Abstract

Fumonisin B1 (FB1) is an important toxin which poses global concerns in terms of food safety. Curcumin (Cur), a natural polyphenolic compound, has strong antioxidant and anti-inflammatory effects. Meanwhile, the mechanisms underlying the mitigation of FB1-induced toxicity by Cur are not fully understood, limiting its potential application as a novel feed additive to prevent FB1 toxicity. In this study, porcine kidney cells (PK-15) were used as an experimental model, utilizing mRNA and miRNA transcriptome technologies. The results revealed that Cur upregulated miR-1249 and inhibited the target gene *Ern1* in the PK-15 cells, thereby suppressing the IRE1/MKK7/JNK/CASPASE3 endoplasmic reticulum (ER) stress pathway and alleviating FB1-induced cell apoptosis. Cell transfection experiments confirmed that Cur effectively attenuated the apoptosis induced by ER stress following transfection with a miR-1249 inhibitor. Similarly, transfection with a miR-1249 mimic alleviated the ER stress and FB1-induced PK-15 cell apoptosis. These findings reveal that Cur mitigates FB1-induced ER stress and significantly reduces apoptotic damage in porcine kidney cells.

## 1. Introduction

Fumonisin B1 (FB1) is a common mycotoxin produced by *Fusarium verticillioides*, *Fusarium oxysporum*, and *Fusarium solani* [1]. This toxin contaminates vegetables and animal products. Contamination rates in maize reach as high as 96.6%, with concentrations of up to 42.1 mg/kg, exceeding the European Commission’s maximum allowable FB concentration of 4.0 mg/kg [2]. Dietary exposure to FB1-contaminated food is a primary source of toxin intake, posing significant health risks to livestock and humans. FB1 is classified under Group 2B carcinogens [3]. FB1 poses a severe threat to the livestock industry. It reduces the nutritional value and affects the sensory characteristics of animal feed, ultimately decreasing feed intake and production performance in livestock. Moreover, FB1 causes poisoning in animals, resulting in substantial economic losses in the livestock industry [4]. Studies report that FB1 causes acute and chronic toxicity in animals, inducing significant renal damage, including glomerular injury, tubular dysfunction, and renal cytotoxicity, accompanied by histopathological changes such as edema and proximal tubular hypertrophy [5,6].

Exposure to external stressors, such as pathogen infections, toxic compounds, hypoxic conditions, ultraviolet radiation, nutritional deficiencies, and mechanical damage, typically activates several complex regulatory mechanisms in the cells to alleviate these stress conditions. Severe stress induces the significant accumulation of misfolded and unfolded proteins in the endoplasmic reticulum (ER) [7]. This triggers the unfolded protein response (UPR) through local ER sensors, including PERK, IRE1, and ATF6, thereby optimizing the cell’s protein-folding capacity and alleviating the stress conditions. However, if the adaptive response fails to restore the ER’s protein-folding function or if the ER stress persists and is severe, the UPR signaling pathway may induce apoptosis [8]. The programmed cell death (PCD) induced by fungal toxins typically involves the activation of members of the caspase family, including caspase-9, caspase-8, and caspase-3 [9]. FB1 indirectly regulates the expression of P53, PUMA, and caspase 3 by inducing oxidative stress and phosphorylating the JNK released from the ER, thereby triggering cell apoptosis [10]. Exposure to 8 μM of FB1 exacerbated kidney damage in mice, leading to a significant reduction in the mitochondrial membrane potential and upregulation of the expression of pro-apoptotic genes [11]. Cell cycle arrest was observed in FB1-treated pig kidney epithelial cells, which enhanced the sensitivity of these cells to apoptosis induced by tumor necrosis factor (TNF) [12]. Exposure of mice to FB1 (2.5 mg/kg) activates ER stress, causing increased apoptosis and autophagy in mouse colonic cells and the elevated expression of IRE1α, p-JNK, Casp3, and LC3I/II [13]. These findings indicate the critical role of FB1-induced ER stress in cell apoptosis. Our previous studies have demonstrated that FB1 induces cell apoptosis, but the molecular mechanisms underlying FB1-induced apoptosis remain unclear [14].

Previous studies have demonstrated that curcumin (Cur), a hydrophobic antioxidant, scavenges free radicals through its electron-donating properties. This effect activates antioxidant response elements and inhibits the oxidative stress caused by reactive oxygen species [15]. Previous findings report that Cur exhibits anti-inflammatory, antioxidant, anti-apoptotic, and anti-tumor activities [16,17,18]. Moreover, Cur is a systemic multi-target drug demonstrating broad-spectrum efficacy across various health conditions [19,20]. Cur can activate multiple survival kinases and transcription factors, including the signaling pathway of PI3K/AKT and JAK2/STAT3, increasing the Bcl-2/Bax ratio and inhibiting apoptotic pathways and cell death [21,22]. Additionally, Cur prevents apoptosis-related heart diseases by inhibiting the expression of NADPH oxidase and ER stress signaling proteins [23]. However, the molecular mechanisms through which Cur antagonizes FB1-induced renal cell injury remain unclear. Therefore, this study investigated whether Cur could alleviate cell apoptosis damage by mitigating FB1-induced ER stress.

miRNAs are essential endogenous non-coding small molecules that play pivotal roles in cellular functions and signaling pathways [24]. In physiological diseases, miRNAs play significant roles in processes such as regulating cell apoptosis, providing a new theoretical basis for disease treatment and prevention [25]. For example, miR-21 and miR-34 family expression is associated with cell apoptosis in tumors [26,27]. Previous studies have reported that fungal toxins can modulate the expression of miRNAs in organismal cells, indicating that miRNAs can be used as markers for toxin exposure and targets for the treatment of toxin poisoning, offering new directions for toxin poisoning research [27,28]. Studies have shown that ochratoxin A (OTA) downregulates the genes of *Nrf2* and *HO-1* expression by inhibiting miR-132 and miR-200c expression, thereby increasing the ROS levels in the body and inducing OTA’s toxic effects [29]. In vivo experiments have demonstrated that zearalenone (ZEA) upregulates the expression of the apoptosis gene *Bad* by activating the expression of miR-1343, miR-331-3p, and miR-744, which downregulate the expression of *Pak4* and *Elk1*, ultimately activating the caspase apoptosis pathway [30]. However, no studies have explored how curcumin mitigates FB1-induced apoptosis damage in cells from the perspective of the miRNA expression profiles.

Therefore, this study selected PK-15 cells as the model system in which to comprehensively investigate the impact of FB1 on the gene expression in porcine kidney cells, with a primary focus on the changes in the expression profiles of genes and miRNAs associated with apoptosis. This comprehensive analysis aimed to reveal the toxic mechanisms of FB1 and provide essential clues for the future development of targeted drugs for the prevention and treatment of FB1 poisoning. Moreover, we investigated the effect of Cur on FB1 toxicity and its potential mechanisms, offering new insights for the treatment of FB1 poisoning.

## 2. Materials and Methods

### 2.1. Cell Culture

The PK-15 cells were obtained from the ATCC cell bank and maintained in our lab. High-glucose DMEM (Procell, Wuhan, China) supplemented with 20% fetal bovine serum (Hyclone, Marlborough, MA, USA) at 37 °C with 5% CO_2_ was used to culture the PK-15 cells. The PK-15 cells were cultured at a concentration of 1 × 10^5^ cells/mL. The cells were detached from the culture flask when the cell density reached 70–80% using trypsin-containing EDTA (Procell, Wuhan, China). The digestion was terminated using a complete culture medium, and the cells were passaged under sterile conditions.

### 2.2. Test Reagents

The FB1 standard (purity > 99%) was supplied by Sourceleaf Technology Co., Ltd. (Shanghai, China). The Cur standard (purity > 98%) was obtained from Solarbio Technology Co., Ltd. (Solarbio, Beijing, China). 4-PBA (Selleck, Shanghai, China, S3592) and 4μ8C (Selleck, China, S7272) are small-molecule inhibitors of IRE1 and ER stress, respectively. The cells were pre-treated with the small-molecule inhibitors for 4 h, washed with PBS, and then treated with FB1 or Cur. The cells in the control group (CON) were cultured under normal conditions for 24 h. PK-15 cells were treated with FB1 (105.87 μmol/L), Cur (30 μmol/L), and a combination of FB1 and Cur for 24 h, corresponding to the FB1 group, the Cur group, and the FB1 + Cur group, respectively.

### 2.3. Sequencing and Analysis

The sequencing experiment comprised two groups, the CON group and the FB1 group, each with three independent replicates. Total RNA was extracted from the cells using Trizol (Takara, Osaka, Japan, Catalog No.: 15596026) for sequencing. The RNA quality and concentration were evaluated to ensure the reliability of the experiment. Library preparation and sequencing were carried out in strict accordance with Illumina’s standard procedures. A TruSeq Small RNA Sample Prep Kit (Illumina, San Diego, CA, USA) was used to prepare the small RNA sequencing library. The Illumina Hiseq2000/2500 was used to sequence the constructed library. The raw data underwent a series of bioinformatics analyses and data processing, including a differential expression analysis of the mRNAs and miRNAs, KEGG and GO enrichment analyses of differentially expressed RNA (OmicShare), a clustering analysis, the generation of volcano plots, and a miRNA–Gene–KEGG interaction analysis.

### 2.4. Prediction of the miRNA Target Genes

Porcine kidney cells were used as the experimental model in this study. The TargetScan and miRanda webservers were used to predict the target genes for significantly differentially expressed miRNAs. The target genes predicted by the two algorithms were filtered based on the software’s scoring criteria. For the TargetScan algorithm, we excluded target genes with a context score percentile below 50. For the miRanda algorithm, target genes with a maximum free energy (Max Energy) greater than -10 were eliminated. The final target genes of the differentially expressed miRNAs were determined by taking the intersection of the predictions from these two software programs.

### 2.5. The Cell Viability Assay Using CCK-8

After achieving confluency, the cells were seeded into a 96-well plate, with each well containing 100 µL of cell suspension. When the cell density reached 70–80%, 10 μL of CCK-8 solution was added to each well (Solarbio, Beijing, China, Catalog No.: 1210), and then the plate was incubated in a cell culture incubator for 2 h, ensuring no bubble formation. After cell incubation, a microplate reader (Thermo, Waltham, MA, USA, Multiskan FC) was used to measure the absorbance, and the cell viability percentage was calculated using the formula Cell Viability (%) = [A (treated) − A (blank)]/[A (untreated) − A (blank)] × 100%. Finally, the data were statistically analyzed using GraphPad Prism (v8.0) software.

### 2.6. Cell Transfection and Target Gene Validation

Cell transfection was conducted using the pmiR-RB-Report^TM^ dual-luciferase reporter vector and the riboFECT CP Transfection Kit (Catalog No.:C10511-05), sourced from Ribobio (Guangzhou, China), according to the manufacturer’s instructions. Firstly, the 3′UTR sequences of the target genes predicted to bind with the miRNAs were amplified and inserted into the dual-luciferase reporter gene vector. Subsequently, the gene vectors were co-transfected into the PK15 cells with miRNA mimics, negative control miRNA mimics, miRNA inhibitors, and a negative control miRNA inhibitor. After 24 h of transfection, the wild-type and mutant plasmid vectors were co-transfected with the miRNA mimic or inhibitor. The fluorescence intensity of the dual-luciferase enzyme’s catalysis was quantitatively measured using a fluorescence microplate reader (Thermo, Multiskan FC, Waltham, MA, USA) to determine the regulatory effects of the miRNAs on the target genes.

### 2.7. Analysis of Cell Apoptosis Using a TUNEL Assay

When the PK-15 cells cultured in the six-well plates achieved 70–80% density, a TUNEL assay kit (Servicebio, Wuhan, China, Catalog No.: G1504) was used to detect apoptosis. Fluorescent images of the cells (100 μm) were observed and captured using a fluorescence microscope (Nikon, Minato, Japan). Each treatment group comprised five independent replicates, corresponding to five random fields of view. The total numbers of cells (blue) and positive cells (green) were quantified using ImageJ 1.53t software to assess the degree of cell apoptosis.

### 2.8. Evaluation of Apoptosis and ROS Content Using a Flow Cytometry Analysis

The cells were cultured in six-well plates, with three independent replicates in each treatment group. The cell concentration in each group was set at 1 × 10^5^ cells/mL. Apoptosis detection was performed using a flow cytometry apoptosis detection kit (Servicebio, China, Catalog No.: G1511), whereas the intracellular ROS levels were determined using a flow cytometry ROS detection kit (Abcam, Cambridge, UK), following the respective manufacturer’s instructions. The flow cytometry analysis of the cells in the different groups was performed using a flow cytometer (Sony, SH800, San Jose, CA, USA). FlowJo and GraphPad software were utilized for the statistical data analysis.

### 2.9. Immunofluorescence Detection of Target Gene Expression

Cells were seeded onto coverslips, fixed with 4% paraformaldehyde for 30 min, and washed. Subsequently, the cells were blocked with 3% bovine serum albumin, washed three times, and then incubated with the primary antibody against IRE1 (Abclone, Wuhan, China, Catalog No.: A21021) overnight at 4 °C, followed by washing and then incubation with the diluted fluorescent secondary antibody (Abclone, Wuhan, China, Catalog No.: A24925) for 30 min. DAPI staining solution was added under dark conditions, and the cells were incubated for 10 min. Images were captured using a laser confocal microscope (Olympus, FV3000, Tokyo, Japan) from 5 randomly selected fields (40 μm). The fluorescence intensity was quantitatively analyzed using ImageJ software, and the statistical analysis was performed using GraphPad software.

### 2.10. Determination of the Cell Proliferation Capability

Cells were cultured into 6-well plates, and their proliferation was assessed using the Cell Proliferation Assay Kit (Servicebio, China, Catalog No.: CA1174). The cell proliferation was visualized under a fluorescence microscope (50 μm), with five random fields selected per cell sample. The analysis was performed using ImageJ software to examine the ratio of Edu-positive cells to DAPI-positive cells. The GraphPad software was used for the statistical analysis.

### 2.11. Validation of Target Gene Expression

Trizol (Takara, Japan, Catalog No.: 15596026) was used to extract total RNA from the cells. The PrimeScript™ RT kit (Takara, Japan, Catalog No.: RR037A) was used to perform reverse transcription of the RNA for the mRNA, and the miRNA 1st Strand cDNA Synthesis Kit (Vazyme, Nanjing, China, Catalog No.: MR101-02) was used for the miRNA. The qRT-PCR reactions for the mRNA and miRNA were conducted using ChamQ Universal SYBR qPCR Master Mix (Vazyme, China, Catalog No.: Q71103) with the following conditions: 95 °C for 30 s, 1 cycle; 95 °C for 10 s, 60 °C for 30 s, 40 cycles; and 95 °C for 15 s, 60 °C for 60 s, 95 °C for 15 s, 1 cycle. The 2^−ΔΔCt^ method was used for the data analysis, and the relative expression level was determined as follows: ΔΔCt = (Ct value of target gene—Ct value of internal reference gene)—(Ct value of blank group gene—Ct value of blank group gene). The gene upstream and downstream primer sequences are shown in Table 1.

### 2.12. Detection of Apoptotic Proteins

The cell samples were washed with PBS buffer (Solarbio, China, Catalog No.: P1020) and cultured with the cell lysis buffer and the protease inhibitor (Solarbio, China). The total cellular proteins were extracted after cell disruption using a cell sonicator (Covaris, S220). The BCA Protein Assay Kit (Solarbio, China, Catalog No.: PC0020) was used to quantify the target proteins following the manufacturer’s instructions. The OD values were measured at 562 nm using a fluorescence microplate reader (Thermo, Multiskan FC), and the protein concentration was calculated. The samples were subjected to denaturation in a PCR machine (Thermo, VeritiPro) at 100 °C for 5 min. Protein samples were loaded into wells containing gel for electrophoresis and transferred and incubated with primary and secondary antibodies. The electrophoresis conditions were set to 120 v for 2 h. The transfer conditions and gel concentration were adjusted based on the molecular weight of the proteins. The primary antibodies against the target proteins were purchased from ABclonal Technology Co., Ltd. The protein bands on the membrane were visualized using an ECL Western Blotting Substrate (Solarbio, China, Catalog No.: PE0010), and the protein quantification and analysis were performed using ImageJ and GraphPad software.

### 2.13. Data Analysis

All of the experimental results in this study were derived from at least three independent replicates and are presented as the mean ± standard deviation ($\bar{x}$ ± *s*). The GraphPad Prism (v8.0) software was used for the statistical analysis. Differences between groups were assessed using the ANOVA method, followed by a post hoc test for pairwise comparisons. Differences were considered statistically significant when *p* < 0.05, indicating significant intergroup differences, with *p* < 0.01 indicating highly significant intergroup differences.

## 3. Results

### 3.1. Curcumin Alleviates FB1-Induced Apoptosis in Porcine Kidney Cells

**Figure 1:**
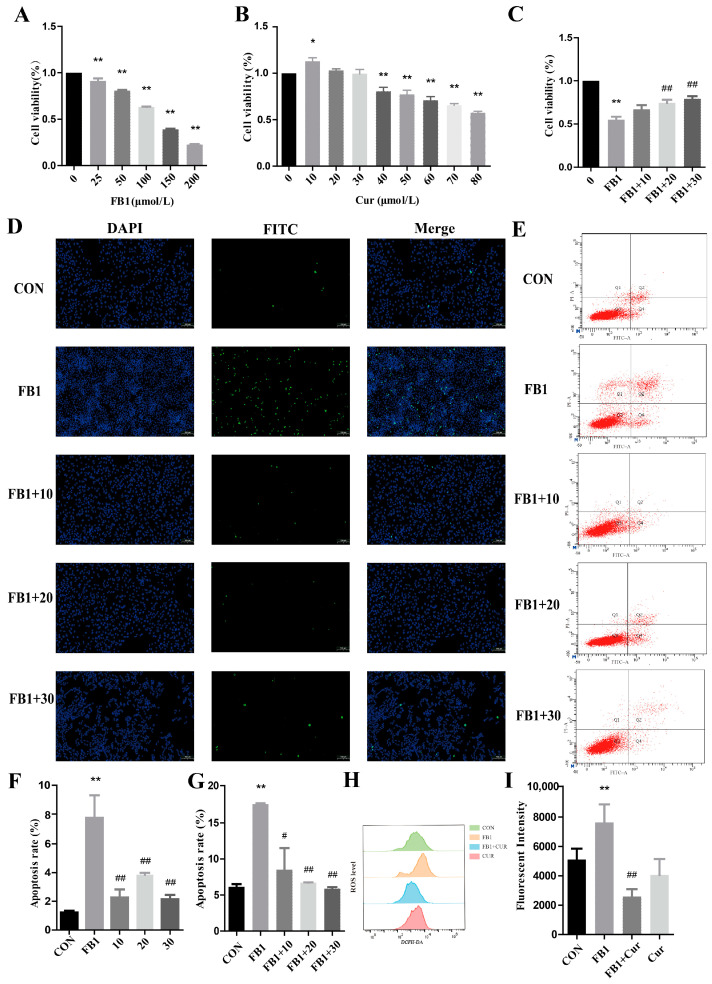
The protective effect of Cur on FB1-induced apoptosis in porcine kidney cells. (**A**–**C**) Effect of FB1, Cur, and their combination on cell viability assessed using CCK assay. (**D**,**F**) Detection of cell apoptosis detected using TUNEL assay (100 μm). DAPI indicates nuclear staining (blue), FITC represents apoptotic cells (green), and Merge denotes the overlap of the images in the first and second columns. (**E**,**G**) Apoptotic rate assessed using flow cytometry. Q1 indicates PI single-stained positive cells, i.e., mechanically damaged cells. Q2 represents Annexin V-FITC and PI double-stained positive cells, i.e., necrotic cells or late-stage apoptotic cells. Q3 denotes live cells. Q4 indicates Annexin V-FITC single-stained positive cells, i.e., early-stage apoptotic cells. (**H**,**I**) Intracellular ROS levels measured using flow cytometry. In the Cur group, the concentration of Cur was 30 μmol/L. * and ** denote significant differences compared to the CON group (*p* < 0.05 and *p* < 0.01, respectively), whereas # and ## denote significant differences compared to the FB1 group (*p* < 0.05 and *p* < 0.01, respectively).

The half-lethal concentration of FB1 for the cells was evaluated using the CCK-8 assay over 24 h. When the FB1 concentrations were 25, 50, 100, 150, and 200 μmol/L, PK-15 cell viability exhibited a dose-dependent decrease with increasing FB1, and the cell viability levels were significantly lower compared to those in the CON group (Figure 1A). The regression analysis using GraphPad Prism software revealed an IC_50_ value of 105.87 μmol/L for FB1. Consequently, this concentration was used as the optimal cytotoxic dose for subsequent experiments. The effect of Cur on porcine kidney cell viability was assessed using the CCK-8 assay over 24 h (Figure 1B). The cell viability significantly increased at 10 μmol/L compared to that in the CON group. However, as the Cur concentrations increased to 40, 50, 60, 70, and 80 μmol/L, the cell viability exhibited a dose-dependent decrease, significantly differing from that in the CON group. Therefore, 10, 20, and 30 μmol/L Cur concentrations were selected for further experiments. The combined effect of FB1 and Cur on the viability of the cells was evaluated using the CCK-8 assay over 24 h (Figure 1C). The results showed a significant decrease in cell viability in the FB1 group compared to that in the CON group. Notably, co-treatment of the cells with FB1 and 10 μmol/L of Cur showed an insignificant increase in cell viability, whereas co-treatment with 20 and 30 μmol/L of Cur significantly increased the cell viability compared to that in the CON group. These findings indicate that 20 and 30 μmol/L of Cur can ameliorate the adverse effects of FB1 on PK-15 cell viability.

The effect of a combination of FB1 and Cur on PK-15 cell apoptosis was investigated using the TUNEL assay (Figure 1D), with the rate of apoptosis illustrated in Figure 1F. Early apoptosis was assessed using flow cytometry (Figure 1E), and the levels of apoptosis are presented in Figure 1G. The two methods yielded consistent results, showing a significant increase in the apoptosis rate in the FB1 group compared to that in the CON group. However, the cells co-treated with FB1 and Cur showed a significant decrease in the apoptosis rate compared to that in the cells exposed solely to FB1.

Changes in the intracellular ROS levels were measured using flow cytometry (Figure 1H), and the fluorescence intensities of the various groups are presented in Figure 1I. The results demonstrated a significant increase in the fluorescence intensity in the FB1 group compared to that in the CON group. Conversely, a combination of FB1 with 30 μmol/L of Cur resulted in a significant decrease in the fluorescence intensity compared to that in the FB1 group. These findings imply that exposure of the cells to FB1 increases ROS levels and ER stress, whereas Cur mitigates the ER stress caused by FB1 in PK-15 cells.

### 3.2. FB1 Modulates the miRNA and mRNA Expression in Porcine Kidney Cells

**Figure 2:**
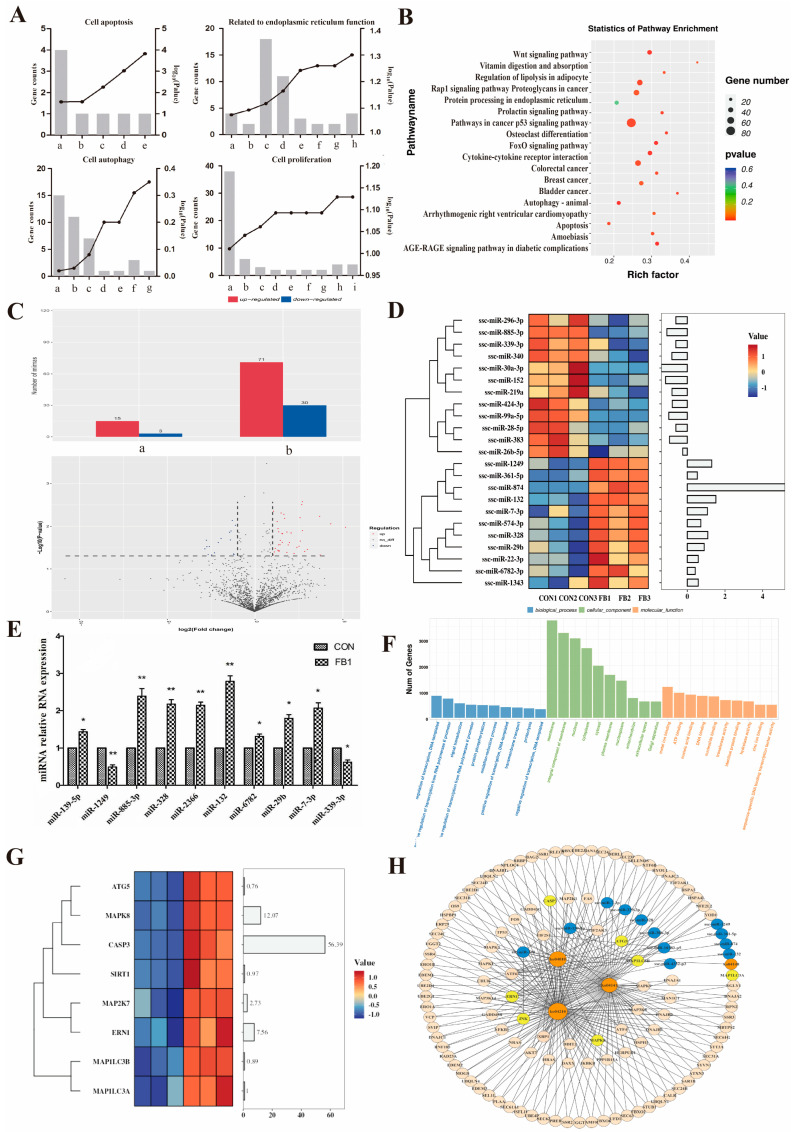
Impact of FB1 on miRNA and mRNA expression levels in porcine kidney cells. (**A**) GO annotation analysis of differentially expressed mRNAs. In the GO apoptosis terms, a, b, c, d, and e represent the initiation phase of apoptosis; cellular component disassembly involved in the initiation phase of apoptosis; negative regulation of cysteine-type endopeptidase activity involved in the initiation of apoptosis; cysteine-type endopeptidase activity involved in the initiation phase of apoptosis; and negative regulation of the initiation phase of apoptosis, respectively. In the GO ER function terms, a, b, c, d, e, f, g, and h represent the ER Golgi intermediate compartment membrane; maintenance of protein localization in the ER; response to ER stress; the ER lumen; protein localization to the ER exit site; regulation of ER-stress-induced intrinsic apoptotic signaling; the rough ER membrane; and protein folding in the ER. In the GO cell autophagy terms, a, b, c, d, e, f, and g represent the regulation of autophagy; macroautophagy; positive regulation of macroautophagy, selective autophagy; positive regulation of chaperone-mediated autophagy; negative regulation of macroautophagy; and negative regulation of mitochondrial autophagy. In the GO cell proliferation terms, a, b, c, d, e, f, g, h, and i represent the regulation of cell proliferation; negative regulation of vascular smooth muscle cell proliferation; negative regulation of osteoblast proliferation; regulation of chorionic trophoblast cell proliferation; positive regulation of the cell proliferation involved in heart morphogenesis; negative regulation of skeletal muscle satellite cell proliferation; osteoclast proliferation; regulation of T-cell proliferation; and cell proliferation in the forebrain. (**B**) KEGG pathway analysis of differentially expressed mRNAs. (**C**) Statistical analysis of differentially expressed miRNAs. The left panel illustrates the frequency distribution of upregulated and downregulated miRNAs, where a represents significantly differentially expressed miRNAs (*p* < 0.01) and b represents differentially expressed miRNAs (*p* < 0.05). The volcano plot on the right presents differentially expressed miRNAs, with each point representing a gene. In the volcano plot, the red dots indicate upregulated genes, blue dots indicate downregulated genes, the *x*-axis represents the fold change in the miRNA expression level between samples, and the *y*-axis represents the statistical significance of the miRNA expression levels. (**D**) Cluster analysis of differentially expressed miRNAs. (**E**) Validation of differentially expressed miRNAs using qRT-PCR. (**F**) Functional analysis of target genes through GO analysis. (**G**) Pathway enrichment analysis of target genes. (**H**) miRNA–gene–KEGG interaction analysis. * and ** denote significant differences compared to the CON group (*p* < 0.05 and *p* < 0.01, respectively).

After the mRNA transcriptome sequencing analysis, differentially expressed mRNAs were functionally annotated through a Gene Ontology (GO) analysis. Enriched GO categories associated with cell apoptosis, ER function, cell autophagy, and cell proliferation were identified (Figure 2A), and the number and levels of differentially expressed genes were statistically analyzed. The results showed that FB1 induced apoptosis and autophagy in the PK-15 cells, affecting ER function and the cell proliferation capacity.

A Kyoto Encyclopedia of Genes and Genomes (KEGG) pathway enrichment analysis was performed to explore the mechanisms underlying the effects of FB1 on the PK-15 cells further (Figure 2B). The significantly enriched signaling pathways included the signaling pathways of AGE-RAGE, Wnt, FoxO, and p53 and the osteoclast differentiation signaling pathway. Additionally, pathways related to apoptosis, autophagy, and protein processing in the ER were enriched, providing insights into the underlying mechanism of FB1’s action on the PK-15 cells.

The differentially expressed miRNAs were assessed between the CON and FB1 groups using the *t*-test method (Figure 2C). The results showed that some miRNAs were upregulated or downregulated in expression, and a volcano plot was drawn to show the differentially expressed miRNAs. In this study, we identified 119 differentially expressed miRNAs, with the expression of 18 miRNAs showing highly significant differences between the FB1 and CON groups (*p* < 0.01). The differentially expressed miRNAs included 15 upregulated and 3 downregulated miRNAs. Notably, the levels of expression for 101 miRNAs exhibited significant differences between the FB1 and CON groups (*p* < 0.05), with 71 upregulated and 30 downregulated miRNAs.

Highly differentially expressed and mature miRNAs (24 miRNAs) were selected from the miRNA expression profile (Figure 2D). Each group comprised three independent replicate samples, and the differential expression of these miRNAs was quantitatively analyzed and presented as bar charts. Subsequently, qRT-PCR was used to validate the levels of expression for selected miRNAs (Figure 2E). The validation results demonstrated the significant upregulation (*p* < 0.05) of miRNA-139-5p, miRNA-6782, miRNA-29b, and miRNA-7-3p and the highly significant upregulation (*p* < 0.01) of miRNA-885-3p, miRNA-328, miRNA-2366, and miRNA-132 in the FB1 group compared to the CON group. Conversely, miRNA-1249 expression was significantly downregulated (*p* < 0.05), whereas miRNA-339-3p was highly significantly downregulated (*p* < 0.01).

TargetScan 7.2 and miRanda 3.3a software was used to predict the target genes of the differentially expressed miRNAs induced by FB1 exposure to explore their biological functions further. The intersection of the results from the two prediction tools was selected as the potential target genes (Figure 2F). A GO analysis was performed on these target genes. The results showed that these target genes were mainly involved in transcriptional regulation, growth maintenance, intracellular signaling, metabolic regulation, and enzyme activity, providing important insights for elucidating these miRNAs’ biological functions further.

In addition, an interaction analysis was conducted for the apoptosis pathway (ko04210), the autophagy pathway (ko04140), the ER stress pathway (ko04141), and the MAPK pathway (ko04010); the miRNAs related to these pathways; and their target genes (Figure 2H). The analysis comprised 12 miRNAs, four signaling pathways, and the associated target genes (Figure 2H). *Ern1* was identified as a target gene, with apoptosis-related genes including *Ern1*, *Map2k7*, *Jnk*, and *Caspase3*. Subsequently, we focused on exploring the regulatory role of miRNA-1249 for the target gene *Ern1*. A cluster analysis of the differentially expressed mRNAs presented in Figure 2G revealed that the expression levels of *Ern1*, *Map2k7*, *Jnk*, and *Caspase3* were significantly upregulated in the FB1 group compared to those in the CON group. Further experiments were conducted to investigate whether Cur could abrogate the cell apoptosis induced by FB1 by targeting the ER stress pathway.

### 3.3. Validation of the miR-1249 Target Gene Ern1

**Figure 3:**
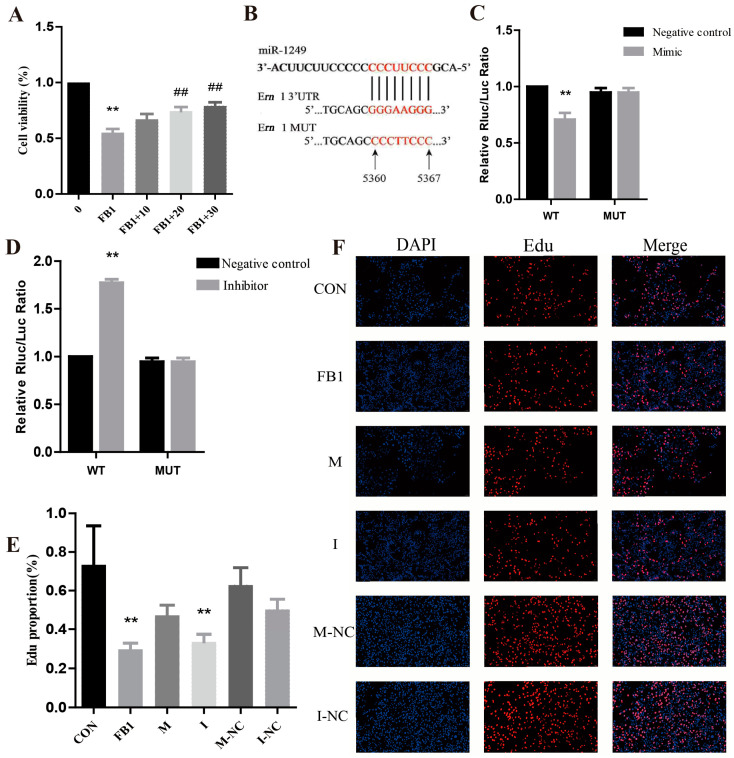
Validation of the predicted target gene (*Ern1*) of miR-1249. (**A**) Expression level of miR-1249 as determined using qRT-PCR. (**B**) Binding sites of miR-1249 with the potential target gene *Ern1*. (**C**,**D**) Luciferase activity for validation of the target gene *Ern1*. (**E**,**F**) Effect of cell transfection on cell proliferation capacity. The analysis comprised six groups: the CON group, the FB1 group, the M group (mimic), the I group (inhibitor), the M-NC group (mimic negative control), and the I-NC group (inhibitor negative control). (**F**) shows the results of Edu staining (50 μm), where DAPI indicates nuclear staining (blue), Edu indicates proliferating cells (red), and Merge represents the overlap of the images from columns 1 and 2. (**E**) presents the statistical results of the positive cell proliferation rate. ** represents a statistically significant difference compared to the CON group (*p* < 0.01). ^##^ represent highly significant (*p* < 0.01) differences compared to FB1 group.

The level of miR-1249 expression was significantly downregulated in the FB1 group after 24 h (Figure 3A). Notably, the level of miR-1249 expression significantly increased after co-treatment with 10, 20, and 30 μmol/L of Cur compared to that in the FB1 group. These results indicate that Cur effectively upregulates the miR-1249 expression in PK-15 cells after FB1 exposure.

The transcriptome sequencing results revealed *Ern1* as a potential target gene for miR-1249. A miR-1249 binding site was predicted in the 3′UTR region of *Ern1*, located between positions 5360 and 5367 (Figure 3B). The luciferase reporter gene assays demonstrated that the luciferase activity in the PK-15 cells significantly decreased after co-transfection with the miR-1249 mimic and the pmiR-RB-Report^TM^ wild-type recombinant plasmid (Figure 3C). Conversely, no significant difference was shown in the luciferase activity after co-transfection with the mutant recombinant plasmid compared to that in the control group. The luciferase activity in the PK-15 cells significantly increased after co-transfection with the miR-1249 inhibitor and the pmiR-RB-ReportTM wild-type recombinant plasmid (Figure 3D). Conversely, no significant difference was observed after co-transfection with the mutant recombinant plasmid compared to that in the negative control group. These findings indicate that *Ern1* is a direct target of miR-1249.

The cell proliferation rates were determined through Edu staining (Figure 3E). The proliferation status of the cells was also evaluated through Edu staining (Figure 3F). The results showed a significant decrease in the cell proliferation rates in the FB1-treated and the inhibitor-treated (I) groups compared to those in the CON group. Notably, the mimic (M), mimic negative control (M-NC), and inhibitor negative control (I-NC) groups showed a reduction in the proliferation rates compared to those in the CON group, although these differences were not statistically significant. These results indicate that treatment of the cells with FB1 promoted the downregulation of miR-1249, ultimately significantly inhibiting the proliferation of the PK-15 cells.

### 3.4. FB1 Activates the Target Gene Ern1 to Induce Apoptosis and ER Stress in PK-15 Cells

**Figure 4:**
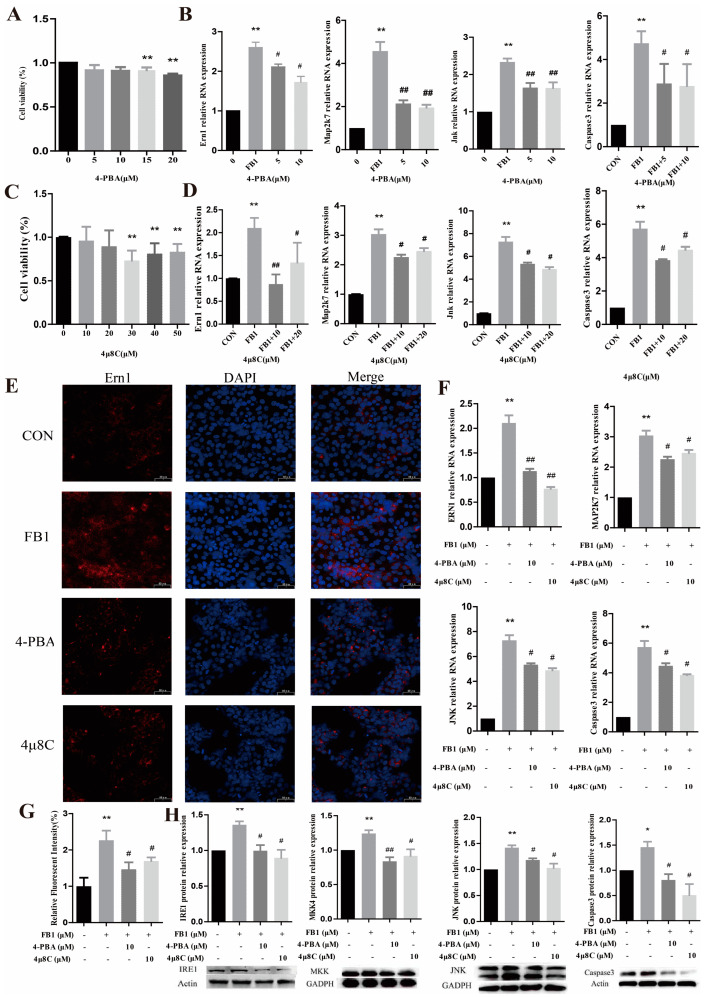
Effect of ER stress on the expression of the target gene *Ern1* and downstream genes. (**A**,**C**) Cell viability levels as determined using the CCK-8 assay to evaluate the effects of small-molecule inhibitors 4-PBA and 4μ8C on the cells exposed to FB1. (**B**,**D**) The influence of the small-molecule inhibitors 4-PBA and 4μ8C on the ER stress pathway as determined using the CCK-8 assay. (**E**,**G**) Immunofluorescence staining results for the target gene (40 μm). DAPI indicates nuclear staining (blue), IRE1 represents positive staining for IRE1, and Merge shows the overlap of the images from the first and second columns. (**F**,**H**) mRNA and protein expression levels of the target genes in the ER stress pathway. * and ** denote significant differences compared to the CON group (*p* < 0.05 and *p* < 0.01, respectively). # and ## indicate significant differences compared to the FB1 group (*p* < 0.05 and *p* < 0.01, respectively).

The optimal dosages of the small-molecule inhibitors 4-PBA and 4μ8C were determined using the CCK-8 assay (Figure 4A,C). A significant decrease in cell viability was observed at concentrations of 15 and 20 μmol/L for 4-PBA and 30, 40, and 50 μmol/L for 4μ8C. Therefore, doses of 5 and 10 μmol/L for 4-PBA and 10 and 20 μmol/L for 4μ8C were used in subsequent experiments to ensure cell viability was not compromised. FB1 significantly upregulated the mRNA expression of *Ern1*, *Map2k7*, *Jnk*, and *Caspase3* in the cells (Figure 4B,D). However, co-treatment with FB1 and 4-PBA or 4μ8C significantly downregulated the expression of *Ern1*, *Map2k7*, *Jnk*, and *Caspase3* compared to their levels in the FB1 group. The optimal dosage for 4-PBA and 4μ8C was determined to be 10 μmol/L based on the results on inhibiting the expression level of the target gene at the mRNA. These findings demonstrate that FB1 activates the ER stress pathway IRE1/MKK7/JNK/CASPASE3. Downregulation of the target gene *Ern1* and a reduction in ER stress levels induced the downregulation of the expression of genes implicated in the ER stress pathway. This finding implies that the downregulation of *Ern1* abrogates the ER stress caused by FB1 in PK-15 cells.

The immunofluorescence analysis of the expression of the IRE1 protein (40 μm) is presented in Figure 4E, and Figure 4G presents the relative fluorescence intensity of the IRE1 protein. These results indicate that FB1 significantly increased the relative fluorescence intensity of the IRE1 protein in the PK-15 cells. However, co-treatment of the cells with FB1 and 4-PBA or 4μ8C significantly decreased the relative fluorescence intensity of the IRE1 protein. These results suggest that reducing the ER stress response in the FB1 group significantly downregulates the level of expression of the IRE1 protein.

FB1 significantly increased the mRNA expression levels of *Ern1*, *Map2k7*, *Jnk*, and *Caspase3* in the cells (Figure 4F). Conversely, treatment of the cells with FB1 and 4-PBA or 4μ8C significantly reduced the mRNA expression levels of *Ern1*, *Map2k7*, *Jnk*, and *Caspase3* (Figure 4F). Analysis of the protein expression results for the target genes showed that FB1 significantly increased the protein expression levels of IRE1, MKK4, JNK, and CASPASE3 in the PK-15 cells (Figure 4H). On the contrary, treatment of the cells with FB1 and 4-PBA or 4μ8C significantly decreased the levels of these target proteins’ expression (Figure 4H).

**Figure 5:**
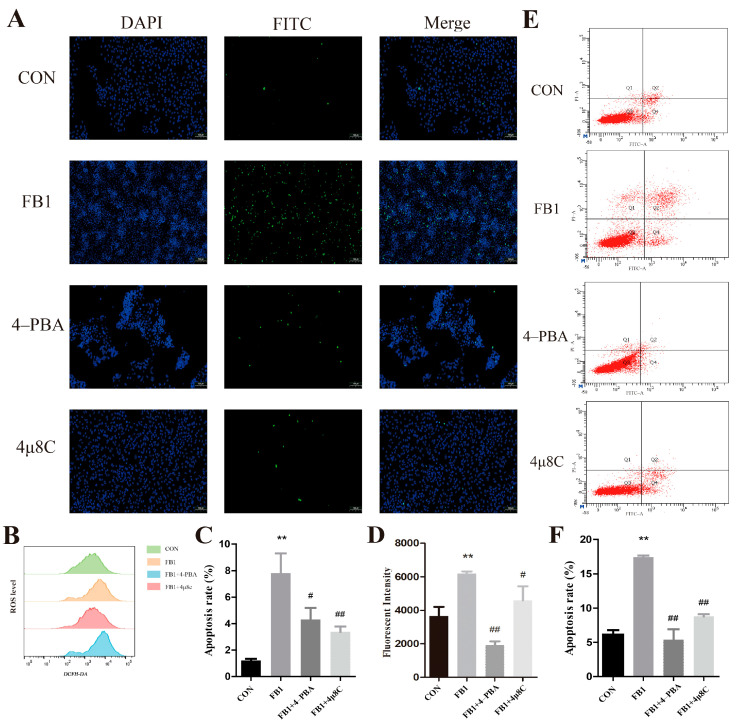
Effects of the *Ern1* gene on apoptosis and endoplasmic reticulum stress. (**A**,**C**) Apoptosis levels as determined using TUNEL assay (100 μm). DAPI represents nuclear staining (blue), FITC represents apoptotic cells (green), and Merge represents the overlap of the results in columns 1 and 2. (**B**,**D**) Intracellular ROS levels measured by using flow cytometry. (**E**,**F**) Apoptosis levels as evaluated using flow cytometry. Quadrant 1 shows cells with Annexin V-FITC and PI positive staining, i.e., necrotic cells or cells in late apoptosis; quadrant 2 indicates nude cells with PI single positive staining, i.e., mechanically damaged cells; quadrant 3 indicates living cells; and quadrant 4 indicates Annexin V-FITC single-stained positive cells, i.e., early apoptotic cells. (**G**,**H**) Intracellular ROS levels as determined using flow cytometry. The concentration of Cur in the Cur group was 30 μmol/L. ** represent highly significant (*p* < 0.01) differences compared to the CON group, respectively; # and ## represent significant (*p* < 0.05) and highly significant (*p* < 0.01) differences compared to the FB1 group, respectively.

A TUNEL assay was conducted to assess the apoptosis status of the PK-15 cells (100 μm) (Figure 5A,C). In addition, flow cytometry was used to evaluate early apoptosis of the cells (Figure 4E,F). The experimental results of these two methods were highly consistent, demonstrating a significant increase in the apoptosis rate in the FB1 group compared to the CON group. However, treatment of the cells with FB1 combined with 4-PBA and 4μ8C significantly decreased the apoptotic rate of the cells compared to that in the group exposed solely to FB1.

A flow cytometry analysis of the intracellular ROS levels was performed using the fluorescence probe DCFH-DA, and the image analysis was conducted using FlowJoX v10.8.1 software (Figure 5B,D). The average fluorescence intensity of the cells significantly increased after incubation of the cells with FB1 for 24 h. Conversely, it significantly decreased after treatment of the cells with FB1 in combination with 4-PBA and 4μ8C compared to that in the FB1 group. These results indicate that exposure to Fumonisin 1 increases the ROS levels within PK-15 cells. However, downregulating the expression of IRE1 in the FB1 group or attenuating the ER stress levels significantly reduced the intracellular ROS production. This finding further confirms the significance of *Ern1* in the ER stress pathway.

### 3.5. Cur Alleviates the Effect of FB1 on the ER Stress Pathway in PK-15 Cells

**Figure 6:**
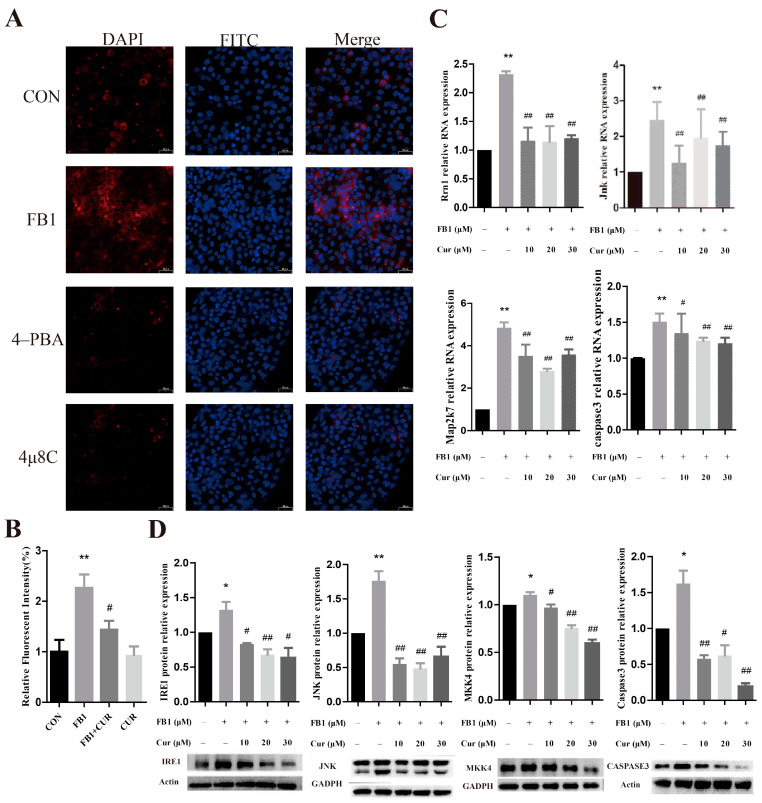
The effect of a combination of FB1 and Cur on the expression profile of genes and proteins related to the ER stress pathway. (**A**,**B**) Immunofluorescence results for IRE1 (40 μm). DAPI represents nuclear staining (blue), IRE1 represents the presence of IRE1, and Merge denotes the overlap of the images from the first and second columns. (**C**,**D**) Effect of Cur on the levels of target gene expression at the mRNA level and the protein level in the ER stress pathway. * and ** indicate significant differences (*p* < 0.05) and extremely significant differences (*p* < 0.01) compared to the CON group, # and ## indicate significant differences (*p* < 0.05) and extremely significant differences (*p* < 0.01) compared to the FB1 group.

The level of expression of the proteins implicated in the ER stress pathway IRE1/MKK7/JNK/CASPASE3 was determined after treatment with a combination of Cur and FB1. The immunofluorescence staining results for IRE1 in the cells are shown in Figure 6A (40 μm), and the relative fluorescence intensity of the IRE1 protein is presented in Figure 6B. Our results indicate a significant increase in the relative fluorescence intensity in the cells treated with FB1, whereas co-treatment with 30 μmol/L of Cur notably decreased the fluorescence intensity. This finding implies that Cur attenuated the upregulation of IRE1 expression induced by FB1.

FB1 significantly increased the mRNA expression levels of *Ern1*, *Map2k7*, *Jnk*, and *Caspase3* in the cells (Figure 6C). However, co-treatment with 10, 20, and 30 μmol/L od Cur significantly decreased the levels of mRNA expression of *Ern1*, *Map2k7*, *Jnk*, and *Caspase3*. Analysis of the protein expression levels of target genes revealed a significant increase in the protein expression levels of IRE1, MKK4, JNK, and CASPASE3 in the PK-15 cells treated with FB1 (Figure 6D). Conversely, co-treatment with 10, 20, and 30 μmol/L of Cur significantly reduced the levels of the target proteins’ expression. In summary, Cur mitigates the ER stress response induced by Fumonisin 1.

### 3.6. Effects of miR-1249 on ER Stress and Apoptosis

**Figure 7:**
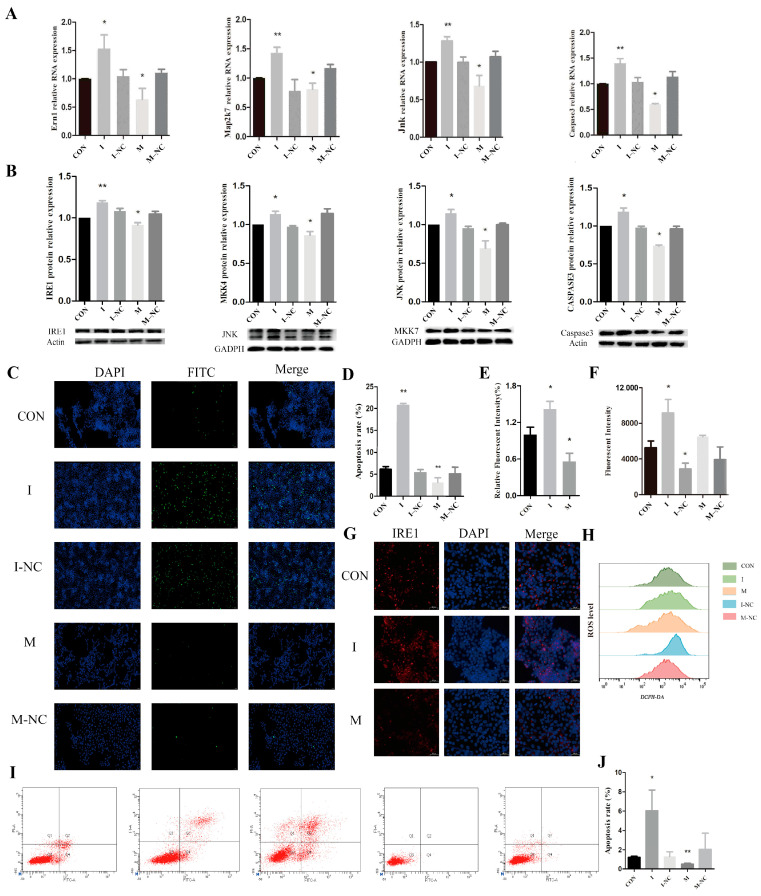
The impact of miR-1249 on ER stress and apoptosis. (**A**,**B**) Effect of miR-1249 on proteins implicated in the ER stress pathway. CON represents the control group, I denotes the miR-1249 inhibitor group, I-NC denotes the miR-1249 inhibitor negative control group, M represents the miR-1249 mimic group, and M-NC denotes the miR-1249 mimic negative control group. (**C**,**D**) Cell apoptotic levels as determined using TUNEL assay (100 μm). DAPI indicates nuclear staining (blue), FITC represents apoptotic cells (green), and Merge indicates the overlap of the images in the first and second columns. (**E**,**G**) Immunofluorescence results for IRE1 (40 μm). DAPI indicates nuclear staining (blue), IRE1 indicates positive staining results for IRE1, and Merge indicates the overlap of the images in the first and second columns. (**F**,**H**) ROS levels in PK-15 cells. (**I**,**J**) Levels of apoptosis in the cells as evaluated using the flow cytometry analysis. The first quadrant represents cells positive for Annexin V-FITC and PI staining, indicating late-stage apoptosis or necrosis; the second quadrant represents cells positive for PI staining only, indicating mechanically damaged cells; the third quadrant represents live cells; and the fourth quadrant represents cells positive for Annexin V-FITC staining only, indicating early-stage apoptosis. * and ** denote significant differences (*p* < 0.05) and highly significant differences (*p* < 0.01) compared to the CON group, respectively.

The PK-15 cells were transfected with a miR-1249 inhibitor (I) and a miR-1249 inhibitor negative control (I-NC), as well as a miR-1249 mimic (M) and a miR-1249 mimic negative control (M-NC), and incubated for 24 h to explore the impact of miR-1249 on the expression profile of genes involved in the IRE1/MKK7/JNK/CASPASE3 pathway. The expression levels of *Ern1*, *Map2k7*, *Jnk*, and *Caspase3* significantly increased in the I group but significantly decreased in the M group compared to those in the CON group (Figure 7A). The expression levels of the *Ern1*, *Map2k7*, *Jnk*, and *Caspase3* genes in the I-NC and M-NC groups did not exhibit significant differences compared to those in the CON group. The immunoblotting results further confirmed these findings, revealing significantly higher expression levels of the IRE1, MKK7, JNK, and CASPASE3 proteins in the PK-15 cells transfected with the miR-1249 inhibitor and significantly lower levels in cells transfected with the miR-1249 mimic compared to those in the CON group (Figure 7B). Notably, no significant differences in the expression levels of the target proteins were observed in the I-NC and M-NC groups compared to those in the CON group. In summary, the downregulation of miR-1249 upregulated the expression of the target gene *Ern1* and its downstream genes, thereby activating the ER stress pathway. Conversely, the upregulation of miR-1249 reduced the level of Ern1 expression and that of its downstream genes, thus inhibiting the ER stress pathway.

The TUNEL assay (Figure 7C,D) and flow cytometry (Figure 7I,J) were conducted to assess the apoptosis level in the PK-15 cells. These two methods yielded consistent results, showing a significantly increased apoptotic rate in group I and a decrease in group M compared to that in the CON group. Notably, no significant differences in the apoptotic rate were found in the I-NC and M-NC groups compared to that in the CON group. These findings imply that the decreased expression of miR-1249 upregulated the expression of *Ern1*, leading to an increase in the apoptosis rate. Conversely, upregulated miR-1249 expression significantly reduced apoptosis by suppressing *Ern1* expression. In summary, *Ern1* plays a crucial role in FB1-induced apoptosis in PK-15 cells.

The immunofluorescence results for the IRE1 expression profile (Figure 7G,E) demonstrated a significant increase in the relative fluorescence intensity in group I compared to that in the CON group, but a significant decrease in the relative fluorescence intensity was observed in group M. This result indicates that miR-1249 significantly affects the expression of the IRE1 protein.

Changes in the intracellular ROS levels are shown in Figure 7F,H. The average fluorescence intensity significantly increased in group I and decreased in group M compared to that in the CON group. Notably, no significant differences in the average fluorescence intensity were observed in the I-NC and M-NC treatment groups compared to that in the CON group. In summary, the downregulation of miR-1249 induced the activation of its target gene *Ern1*, resulting in increased ROS levels and the activation of ER stress in the PK-15 cells. Conversely, the upregulation of miR-1249 significantly reduced the intracellular ROS levels by inhibiting *Ern1* expression.

### 3.7. Cur and the miR-1249 Mimic Suppress Ern1-Induced ER Stress and Cell Apoptosis

**Figure 8:**
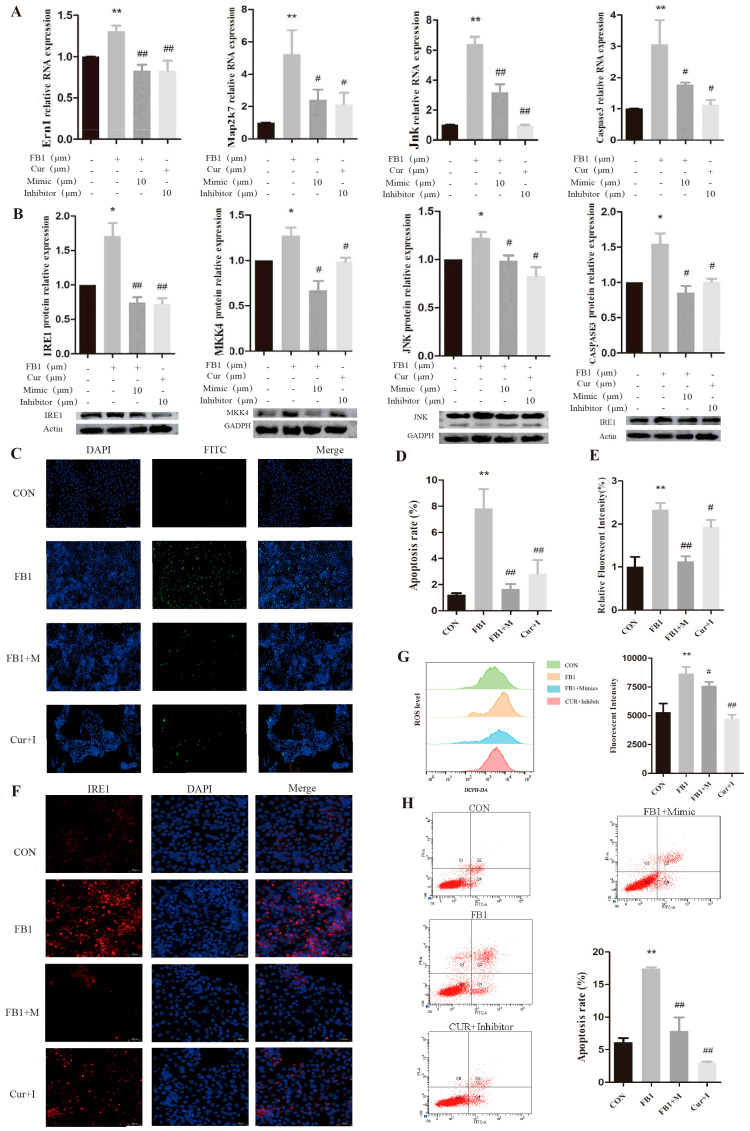
Curcumin and the miR-1249 mimic inhibit *Ern1*-induced ER stress and cell apoptosis. (**A**,**B**) Expression of the target genes at the mRNA and protein levels in the ER stress pathway. (**C**,**D**) Cell apoptosis as assessed using TUNEL assay. DAPI indicates nuclear staining (blue), FITC indicates apoptotic cells (green), and Merge indicates the overlap of the images in columns 1 and 2. (**E**,**F**) Immunofluorescence results for IRE1 (40 μm). DAPI indicates nuclear staining (blue), IRE1 indicates positive staining results for IRE1, and Merge indicates the overlap of the images in the first and second columns. (**G**) Intracellular ROS levels evaluated using flow cytometry. (**H**) Level of apoptosis in the cells determined using flow cytometry (scale bar: 100 μm). * and ** indicate significant differences compared to the CON group (*p* < 0.05 and *p* < 0.01, respectively), # and ## indicate significant differences compared to the FB1 group (*p* < 0.05 and *p* < 0.01, respectively).

Cells exposed to FB1 were transfected with the miR-1249 mimic (the FB1+M group) to explore the role of miR-1249 in the IRE1/MKK7/JNK/CASPASE3 signaling pathway further. In addition, cells transfected with the miR-1249 inhibitor were incubated with Cur (the Cur+I group). The mRNA expression levels of *Ern1*, *Map2k7*, *Jnk*, and *Caspase3* were significantly higher in the FB1 group compared to those in the CON group (Figure 8A). However, the expression levels of *Ern1*, *Map2k7*, *Jnk*, and *Caspase3* were markedly lower in the FB1+M and Cur+I groups compared to those in the FB1 group. The immunoblotting results and the levels of target protein expression are illustrated in Figure 8B. The expression levels of the IRE1, MKK7, JNK, and CASPASE3 proteins were significantly higher in the FB1 group compared to those in the CON group (Figure 8B). Conversely, the expression levels of the IRE1, MKK7, JNK, and CASPASE3 proteins were lower in the FB1+M and Cur+I groups compared to those in the FB1 group. These results imply that the miR-1249 mimic upregulated miR-1249 in the FB1-treated cells, leading to reduced *Ern1* expression and inhibition of the ER stress pathway. These findings and the results presented in Section 3.6 indicate that Cur upregulated the miR-1249 expression in the cells, leading to decreased *Ern1* expression and inhibition of the ER stress pathway.

Cell apoptosis was assessed using the TUNEL assay (Figure 8C,D) and flow cytometry (Figure 8H). These two methods yielded similar apoptotic outcomes. The results showed a significantly higher apoptosis rate in the FB1 group compared to that in the CON group. However, the apoptosis rate was significantly lower in the FB1+M and Cur+I groups compared to that in the FB1 group. These findings imply that the upregulation of miR-1249 expression in the FB1-treated cells effectively mitigated FB1-induced apoptosis. Notably, Cur attenuated the cell apoptosis induced by low miR-1249 expression.

The immunofluorescence results for IRE1 (40 μm) are presented in Figure 8E,F. The FB1 group showed a significantly higher relative fluorescence intensity compared to that in the CON group. Conversely, the relative fluorescence intensity was significantly lower in the FB1+M and Cur+I groups compared to that in the FB1 group. These results indicate that miR-1249 significantly modulated the level of expression of the IRE1 protein.

Changes in the intracellular ROS levels were assessed using flow cytometry (Figure 8G). The FB1 group showed a significantly higher average fluorescence intensity compared to that in the CON group. Conversely, the FB1+M and Cur+I groups showed significantly lower average fluorescence intensities compared to that in the FB1 group. These findings indicate that the upregulation of miR-1249 expression effectively reversed the elevated ROS levels in the FB1-treated cells, thereby alleviating FB1-induced ER stress. Furthermore, these results demonstrated that Cur inhibited the ROS production in the cells through miR-1249 downregulation, leading to decreased ER stress levels. This finding further confirms the importance of ER stress in cell apoptosis.

## 4. Discussion

Cellular homeostasis is disrupted when cells are exposed to external stimuli such as hypoxia, starvation, oxidative damage, pH changes, calcium depletion, low glucose, ATP depletion, and viral infection. This disruption leads to the accumulation of misfolded or unfolded proteins, the release of calcium, the activation of Caspase-12, and the subsequent induction of ER stress [31,32,33]. In vivo and in vitro exposure to FB1 disrupts the redox system, as evidenced by increased ROS levels, lipid peroxidation, decreased levels of the antioxidant glutathione (GSH), and increased levels of oxidative indicators such as GPx and superoxide dismutase (SOD) [34,35,36]. This finding is consistent with ours, where FB1 exposure was able to increase the ROS levels and elevated endoplasmic reticulum stress in the PK-15 cells.

A conservative signaling pathway known as the unfolded protein response (UPR) has evolved to mitigate ER stress in cells. This signaling pathway triggers cell apoptosis to restore ER homeostasis [37,38]. The UPR promotes the expression of specific stress sensor proteins, such as glucose-regulated protein 78 (GRP78/Bip). Molecular chaperones assist in the repair of misfolded and unfolded proteins through GRP78/Bip, promoting protein refolding and degradation to restore cellular homeostasis [32,39]. IRE1 is the most conserved protein among the UPR sensors. In response to the accumulation of unfolded proteins in the ER, IRE1 undergoes trans-autophosphorylation and oligomerization, leading to structural changes in its ribonuclease (RNase) domain. IRE1 splices the X-box-binding protein 1 (XBP-1) mRNA, and the spliced form, XBP-1s, together with other transcription factors, induces the expression of ER-stress-related genes, facilitating the recycling of unfolded proteins to the ER and restoring ER homeostasis [40,41,42]. Consequently, IRE1α promotes mRNA degradation by modulating IRE1α-dependent decay (RIDD), thereby suppressing the synthesis of new polypeptide chains and inhibiting ER stress [43]. However, if the adaptive response fails to restore the protein-folding capacity of the ER or if the ER stress persists, the UPR signal induces cell apoptosis [44,45,46,47]. In addition to splicing XBP1, IRE1 activates JNK and interacts with Caspase-12, indicating that the IRE1-ASK1-JNK axis is crucial in promoting apoptosis [48,49]. In this study, FB1 triggered a cascade of signaling events in the PK-15 cells through the activation of IRE1, leading to a series of signal transduction events, including the activation of MKK7 and the phosphorylation of JNK. These events ultimately result in the activation of *Caspase3*, a key protein that modulates cell apoptosis, playing a crucial role in regulating apoptosis.

A previous study demonstrated that treating liver cancer cells with FB1 at a concentration of 50 μM for 24 h significantly increased the intracellular ROS levels, accompanied by a notable upregulation in the expression of the ER-stress-related proteins PERK and IRE1 and the autophagy marker LC3I/II [36]. Aflatoxin B1 (AFB1), a fungal toxin, upregulates the expression of Chop, GRP78, p-IRE1/Xbp1s, and p-PERK/p-EIF2a in mouse hippocampal neuronal cells, leading to elevated ER stress and apoptosis levels [50]. The T-2 toxin activates the IRE1-JNK and PERK-ATF4-CHOP signaling pathways in goat endometrial epithelial cells, resulting in ER stress and an increased apoptosis rate [51]. Furthermore, IRE1α promotes cell apoptosis through the TRAF2-mediated signaling pathway [52,53]. These findings further highlight the potential mechanism underlying the role of fungal toxins in inducing apoptosis through the ER stress pathway. In our study, we established an FB1 exposure model to study the role of ER stress in FB1-induced apoptotic toxicity further. A significant increase in the ROS levels in the PK-15 cells was shown, indicating elevated oxidative stress levels and activation of the ER stress pathway IRE1/MKK7/JNK/CASPASE3 in the FB1 group. Moreover, treating the FB1-treated cells with Cur, 4-PBA, or 4μ8C downregulated the *Ern1* expression and decreased the ER stress levels in the PK-15 cells. These effects demonstrate the crucial role of ER stress in apoptosis induced by FB1. These findings reveal that Cur abrogates FB1-induced apoptotic toxicity by lowering the ER stress levels in cells, providing important insights revealing more about the regulatory role of ER stress in apoptosis induced by FB1.

Cur elevates the glutathione (GSH) levels in brain cells, reduces the production of ROS under oxidative stress, decreases malondialdehyde (MDA) levels, and inhibits lipid peroxidation [54]. In a previous study, quails exposed to 30 mg/kg of FB1 exhibited a response in their nuclear xenobiotic receptors (NXRs) and a cytochrome P450 system response, attributed to increased ER stress, mitochondrial damage, and the upregulated expression of IL-1β, IL-6, and IL-8 in the kidneys, ultimately leading to renal dysfunction [55]. In addition to its potent anti-inflammatory and antioxidant effects, Cur protects the myocardial cells from apoptosis-related damage by inhibiting the expression of NADPH oxidases (NOXs) and ER signaling proteins [56]. Endoplasmic reticulum stress activates the CAMKII/JNK signaling pathway, ultimately inducing the production of NADPH oxidases (NOXs) and hydrogen peroxide (H₂O₂) [57]. NOXs are membrane proteins expressed in most tissues in the body and are direct sources of reactive oxygen species (ROS). NOXs facilitate the NADPH-dependent single-electron reduction of molecular oxygen into superoxide anions. Consequently, they are ideal targets for alleviating oxidative stress, apoptosis, inflammation, and fibrosis and treating tumors. These mechanisms suggest that curcumin (Cur) is a potentially effective agent for inhibiting ER-stress-induced apoptosis [58,59,60]. In this study, we observed that Cur effectively downregulated the expression of *Ern1* by targeting it, thus reducing the ROS levels in the PK-15 cells, inhibiting the ER stress pathway IRE1/MKK7/JNK/CASPASE3, and significantly suppressing cellular apoptosis. These findings further highlight the potential of Cur in alleviating cellular stress and protecting cells against FB1 toxicity, providing important theoretical evidence for its potential application as a candidate drug for treating ER-stress-related diseases.

miRNAs play crucial roles in physiological, pathological, and toxicological processes, modulating the expression of over half of the proteins in the human body, and have significant potential applications in drug development [61,62]. Early diagnosis of diseases caused by mycotoxins primarily focuses on protein-level studies, whereas miRNAs have not been extensively studied as potential diagnostic markers. Previous studies have shown that mycotoxins can alter the expression of the miRNAs in host cells. OTA upregulated the miR-122 expression in mouse spermatocyte cells (GC-2), thereby inhibiting the expression of *Bcl-w*, activating *Caspase-3*, and ultimately inducing cell apoptosis [63]. AFB1 significantly upregulates the miR-34a expression in HepG2 cells. The upregulation of miR-34a leads to decreased levels of expression of *β-catenin*, *c-myc*, and *Cyclin D1,* which are implicated in the Wnt signaling pathway. These effects result in cell cycle arrest at the S phase and an increased risk of hepatocellular carcinoma (HCC) [64]. Therefore, in-depth studies of the interaction between miRNAs and their target genes are essential for understanding the regulatory mechanisms of miRNAs in pathogenic signaling pathways. In addition, these findings provide a theoretical basis for the prevention and treatment of mycotoxin-related diseases [65].

In the present study, GO and KEGG enrichment analyses revealed that the differentially expressed genes in the FB1 group were enriched in processes such as cell apoptosis, ER function, cell autophagy, and cell proliferation. The significantly differentially expressed mature miRNAs were evaluated, and miR-1249 and its target gene *Ern1* were ultimately selected for the subsequent analysis in this study. The experimental results demonstrated that exposure to FB1 decreased the expression of miR-1249 in the PK-15 cells, leading to increased ER stress levels, activation of the target gene *Ern1*, and a higher rate of cellular apoptosis. Similar results were found in the cells transfected with the miR-1249 inhibitor. The transfection experiments showed that upregulating miR-1249 in the FB1-treated cells or adding Cur to cells with low miR-1249 expression effectively downregulated the expression of *Ern1*, thereby inhibiting the cellular apoptosis induced by ER stress. This finding is significant for exploring the application of Cur in alleviating ER-stress-related diseases. In addition, this finding provides a theoretical basis for using Cur to prevent and treat FB1 toxicity. It may also offer new research directions for studying the mechanisms underlying the toxicity of other mycotoxins.

This study demonstrates that FB1 activates ER stress in PK-15 cells, leading to the downregulation of miR-1249 and the subsequent activation of its target gene *Ern1*. This activation further triggers the ER stress pathway IRE1/MKK7/JNK/CASPASE3, resulting in increased apoptosis of the PK-15 cells. However, the application of Cur reduces the ER stress, upregulating miR-1249 in FB1-treated cells. Consequently, the expression of *Ern1*, the target gene of miR-1249, is suppressed, mitigating the significant apoptotic damage caused by FB1 in PK-15 cells. We also evaluated the intervention effect of Cur in the FB1 toxicity model and explored the regulatory role of miR-1249 and its target gene *Ern1* in the ER stress pathway. These findings indicate the potential application of Cur as a novel feed additive for preventing FB1 toxicity and offer new insights for further research in related fields.

## Figures and Tables

**Table 1:** Gene upstream and downstream primer sequences.

Gene	Primer Sequence (5′-3′)	Accession
*Ern1*-F	ACCGTGGTGTCTCAGGATGTGG	XM_005668695.3
*Ern1*-R	CCAGCCAATGAGCAGGAAGGTG	
*Map2k7*-F	GACTCCATTGCCAAGACCAGAGATG	XM_005661260.3
*Map2k7*-R	GCCAGTTCGTACAGTGTGATCCC	
*Jnk*-F	ACTACAGAGCACCTGAGGTCATCC	XM_021073086.1
*Jnk*-R	ATTTCTCCCATAATGCACCCCACAG	
*Caspase3*-F	AGAATTGGACTGTGGGATTGAGACG	NM_214131.1
*Caspase3*-R	GCCAGGAATAGTAACCAGGTGCTG	
*Gadph*-F	GGCTGTGGGCAAGGTCATCC	NM_001206359.1
*Gadph*-R	TCTCCAGGCGGCAGGTCAG	
miR-1249-F	AGTGCAGGGTCCGAGGTATTTATATACGCCCTTCCCCCCCT	MIMAT0025385
miR-1249-R	ATCCAGTGCAGGGTCCGAGG	
U6-F	TCGCTTTGGCAGCACCTAT	
U6-R	AATATGGAACGCTTCGCAAA	

Note: F stands for forward, and R stands for reverse.

## Data Availability

The raw data necessary to support the results of this article will be promptly made available by the authors upon request.
